# Supplementary material for: Implementation of Modern Therapeutic Drug Monitoring and Lipidomics Approaches in Clinical Practice: A Case Study with Colistin Treatment
Source: Pharmaceuticals (Basel). 2024 Jun 7;17(6):753. doi: 10.3390/ph17060753 (PMC11206893; doi:10.3390/ph17060753)
Supplement: Supplementary file 1 [file pharmaceuticals-17-00753-s001.zip › Pharmaceuticals-3027108-Supplementary material.pdf]

pharmaceuticals

Article

Implementation of Modern Therapeutic Drug Monitoring and Lipidomics Approaches in Clinical Practice: A Case Study with Colistin Treatment

Ivana Gerhardtova [1,2], Ivana Cizmarova [3,4], Timotej Jankech [1,2], Dominika Olesova [1,5], Josef Jampilek [1,2], Vojtech Parrak [1,6], Kristina Nemergutova [6], Ladislav Sopko [6], Juraj Piestansky [1,4,7,*] and Andrej Kovac [1,8,*]

[1] Institute of Neuroimmunology, Slovak Academy of Sciences, Dubravska cesta 9, 845 10 Bratislava, Slovakia; ivka.gerhardtova@gmail.com (I.G.); timotej.jankech@gmail.com (T.J.); dominika.olesova@savba.sk (D.O.); josef.jampilek@gmail.com (J.J.); vojtech.parrak@savba.sk (V.P.)
[2] Department of Analytical Chemistry, Faculty of Natural Sciences, Comenius University Bratislava, Ilkovicova 6, 842 15 Bratislava, Slovakia
[3] Department of Pharmaceutical Analysis and Nuclear Pharmacy, Faculty of Pharmacy, Comenius University Bratislava, Odbojarov 10, 832 32 Bratislava, Slovakia; ivana.cizmarova@fpharm.uniba.sk
[4] Toxicological and Antidoping Center, Faculty of Pharmacy, Comenius University Bratislava, Odbojarov 10, 832 32 Bratislava, Slovakia
[5] Institute of Experimental Endocrinology, Biomedical Research Center SAS, Dubravska cesta 9, 845 10 Bratislava, Slovakia
[6] Clinic of Hematology and Transfusiology, St. Cyril and Methodius Hospital, Antolska 11, 851 07 Bratislava, Slovakia; kika.nemergutova@gmail.com (K.N.); sopko.ladislav@gmail.com (L.S.)
[7] Department of Galenic Pharmacy, Faculty of Pharmacy, Comenius University Bratislava, Odbojarov 10, 832 32 Bratislava, Slovakia
[8] Department of Pharmacology and Toxicology, University of Veterinary Medicine and Pharmacy in Kosice, Komenského 68/73, 041 81 Kosice, Slovakia
* Correspondence: piestansky@fpharm.uniba.sk (J.P.); andrej.kovac@savba.sk (A.K.)

Citation: Gerhardtova, I.; Cizmarova, I.; Jankech, T.; Olesova, D.; Jampilek, J.; Parrak, V.; Nemergutova, K.; Sopko, L.; Piestansky, J.; Kovac, A. Implementation of Modern Therapeutic Drug Monitoring and Lipidomics Approaches in Clinical Practice: A Case Study with Colistin Treatment. *Pharmaceuticals* **2024**, *17*, x. https://doi.org/10.3390/xxxxx

Academic Editor: Sabina Lachowicz-Wiśniewska

Received: 9 May 2024
Revised: 2 June 2024
Accepted: 4 June 2024
Published: 7 June 2024

Copyright: © 2024 by the authors. Submitted for possible open access publication under the terms and conditions of the Creative Commons Attribution (CC BY) license (https://creativecommons.org/licenses/by/4.0/).

(Supplementary material)

Table of content:

Figure S1: Chemical structure of colistin A (polymyxin E_1) and colistin B (polymyxin E_2).

Figure S2: A complex overview of concentration changes in selected individual lipids during the hospitalization of a critically ill patient and his treatment with multiple ATBs including colistin.

Table S1. Concentration levels of individual lipids determined during the colistin therapy.

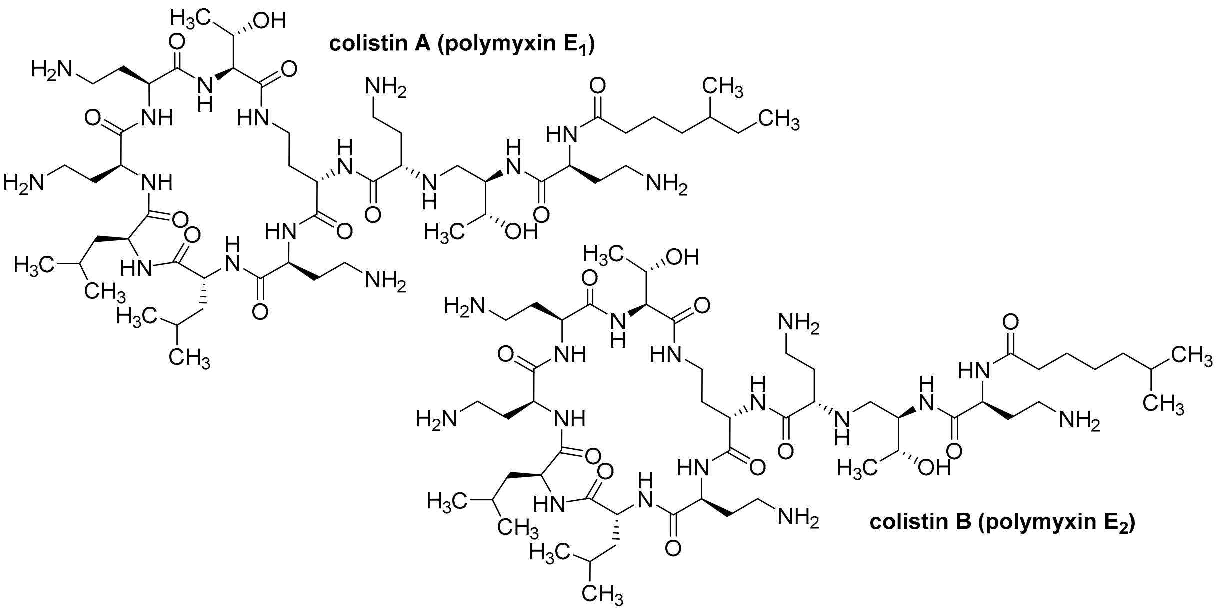

Figure S1. Chemical structure of colistin A (polymyxin E_1) and colistin B (polymyxin E_2).

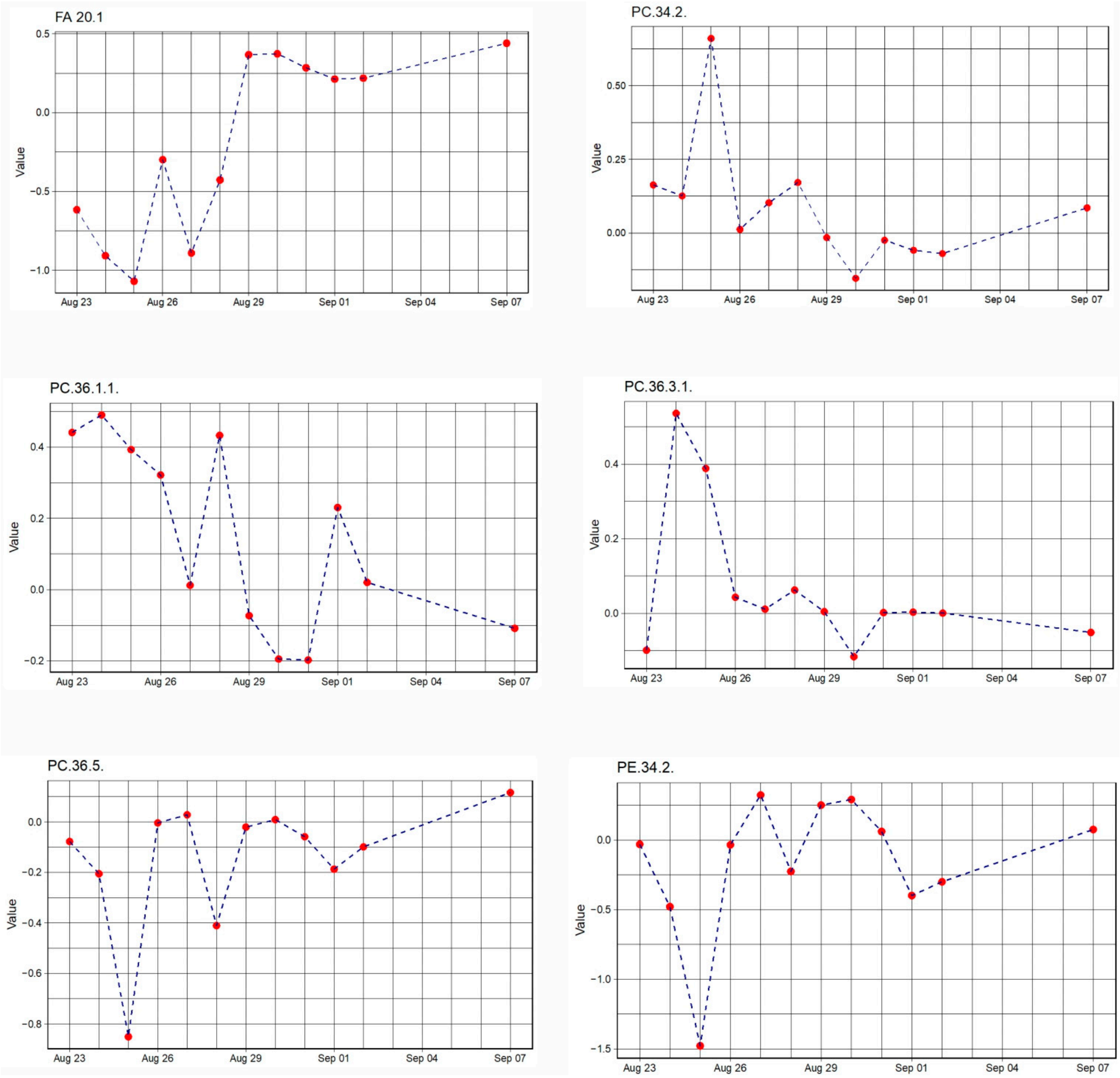

Figure S2. A complex overview of concentration changes in selected individual lipids during the hospitalization of a critically ill patient and his treatment with multiple ATBs including colistin.
